# Crystals lens deposits: a rare occurrence in Bietti corneo-retinal dystrophy

**DOI:** 10.11604/pamj.2022.43.7.34356

**Published:** 2022-09-06

**Authors:** Jihene Sayadi, Manel Mekni

**Affiliations:** 1 Hedi Rais Institute of Ophthalmology, Tunis El-Manar University, Tunis, Tunisia

**Keywords:** Bietti crystalline dystrophy, crystals lens deposits, retinal degeneration

## Image in medicine

Bietti corneo-retinal dystrophy (BCD) is a rare and potentially blinding autosomal recessive disease associated with CYP4V2 mutations. It is characterized by typical progressive chorio-retinal degeneration and crystals deposits in the retina. Crystals deposits in the corneal limbus are described in about one-third of patients. Bietti corneo-retinal dystrophy commonly manifests itself between the second and the third decade. We present a 58-year-old woman with a long history of bilateral progressive loss of vision and nyctalopia. Medical history was unremarkable excluding cystinosis, oxalosis and gyrate atrophy. Visual acuity was 0.1 logMAR in both eyes. Anterior segments examination revealed cortical cataracts and multiple white-yellow glistening crystals lens in both eyes. Deposits were not observed in the corneal limbus. Dilated fundus examination showed multiple and diffuse reflective yellow deposits in the posterior pole and in the mid-peripheral retina. Geographic areas of atrophy of the retinal pigment epithelium-choriocapillaris were present in the posterior pole, associated with areas of hyperpigmentation. Based on these findings, a clinical diagnosis of BCD was established. Spectral domain optical coherence tomography revealed hyperreflective deposits involving all retinal layers and outer retinal tubulations in both eyes (A, B, C). Only few cases of crystal lens deposits in patients with BCD have been published. As crystals lens deposits were described in cases with severe chorio-retinal atrophy and a long follow-up before the crystals could be identified, it was suggested that they would be a trait of advanced disease.

**Figure 1:**
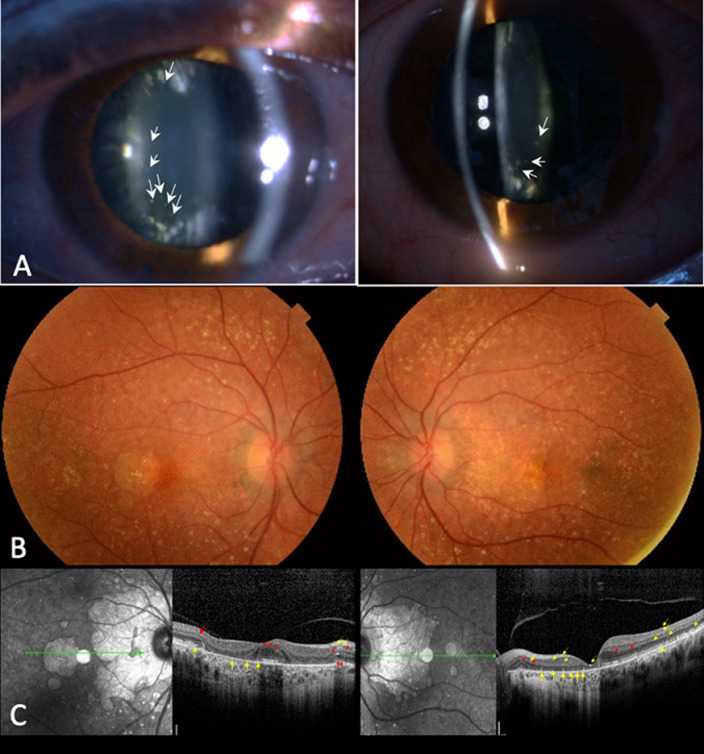
Bietti corneo-retinal dystrophy: A) anterior segment photographs showing glistening yellow-white crystals deposits in the anterior and posterior lens cortex (white arrows); B) color fundus photographs showing multiple, glistening yellow retinal crystals deposits in the posterior pole to the midperiphery of the retina associated with subsequent retinal pigment epithelium (RPE) and choriocapillaris atrophy in both eyes; C) SD-OCT showing retinal crystals deposits as hyperreflective dots involving all retinal layers mainly at the level of the RPE-choriocapillaris complex (yellow arrows) and outer retinal tubulations (red arrows)

